# Socioeconomic gradient in COVID-19 vaccination: evidence from Israel

**DOI:** 10.1186/s12939-021-01566-4

**Published:** 2021-11-08

**Authors:** Mor Saban, Vicki Myers, Shani Ben-Shetrit, Rachel Wilf-Miron

**Affiliations:** 1 Gertner Insititute of Epidemiology & Health Policy Research, Sheba Medical Center, Emek Dotan 1, 5262100 Ramat Gan, Israel; 2 The Buchmann Faculty of Law, Tel Aviv University, Tel Aviv, Israel; 3 School of Public Health, Sackler Faculty of Medicine, Tel Aviv University, Tel Aviv, Israel

## Abstract

**Background:**

Low socioeconomic status (SES) groups have been disproportionately affected by the COVID-19 pandemic. We aimed to examine COVID-19 vaccination rate by neighborhood SES and ethnicity in Israel, a country which has achieved high vaccination rates.

**Methods:**

Data on vaccinations were obtained from the Israeli Ministry of Health’s open COVID-19 database, for December 20, 2020 to August 31, 2021. Correlation between vaccination rate and neighborhood SES was analyzed. Difference in vaccination rate between the first and second vaccine dose was analyzed by neighborhood SES and ethnicity.

**Findings:**

A clear socioeconomic gradient was demonstrated, with higher vaccination rates in the higher SES categories (first dose: r = 0.66; second dose: r = 0.74; third dose: r = 0.92). Vaccination uptake was lower in the lower SES groups and in the Arab population, with the largest difference in uptake between Jewish and Arab localities for people younger than 60, and with the gap widening between first and third doses.

**Conclusions:**

Low SES groups and the Arab ethnic minority demonstrated disparities in vaccine uptake, which were greater for the second and third, compared with the first vaccine dose. Strategies to address vaccination inequity will need to identify barriers, provide targeted information, and include trust-building in disadvantaged communities.

## Background

Low socioeconomic status (SES) groups have been disproportionately  affected by the COVID-19 pandemic, with both the direct effects of higher morbidity and mortality from the virus, as well as the economic consequences of public health restrictions, including lockdowns [1]. Ecological studies show greater COVID-19 burden in lower socioeconomic areas. For example the National Health and Nutrition Examination Survey (NHANES) found disproportionate deaths occurring in minority groups, individuals with below median income, and those with less than high school education [2].

In December 2020, 12 months after the first cases were diagnosed, the COVID-19 Marmot Review suggested that countries which began the pandemic with significant inequality had higher mortality rates [3]. Widening inequalities in living conditions between individuals, communities and regions have generated inequalities in health, including vulnerability to the burden of COVID-19 disease [3].

Vaccines are now available predominantly in wealthier countries, with slower rollout in poorer countries. A successful vaccination campaign requires three things: vaccine supplies (and appropriate storage and distribution), people to implement them (vaccinators), and willingness of people to be vaccinated [4].

It is important to ensure that all groups have access to the vaccine, especially disadvantaged groups who are more susceptible to infection from COVID-19 and at greater risk of severe morbidity and mortality. Where vaccines are available, it remains to be seen whether those in greatest need and most affected by the pandemic will be willing and able to access the vaccine.

Israel rolled out vaccination with the Pfizer BioNTech vaccine on December 20, 2020. In the first weeks, the elderly (60+) and people with health risk factors were invited to receive the vaccine. By early February 2021, vaccination was opened up to all population groups (16+). By March 15, 2020, 55 and 46% of the total population (9.3 million) had received the first and second vaccine doses, respectively. Approximately 90% of people aged over 50 were immune (vaccinated or recovered) by that time [5]. The leading factors in this rapid vaccine rollout and exceptionally high coverage were the organizational, information technology and logistical capacities of Israel’s community-based health care providers (sick-funds) [6]. A media campaign, led by well-known, senior health professionals and celebrities, called on the public to vaccinate. The MOH designed a “green pass” incentive, aimed to “compensate” for the months of social restrictions. This certificate came into effect from February 21, 2021 and allows access to social, cultural and sports events, as well as gyms, hotels and restaurants for those with immunity – whether recovered from COVID-19 or 1 week following the second vaccine dose [7].

Israel has a relatively high Gini coefficient of 36.9, representing a high level of inequality between groups. The population of 9.1 million is made up of a majority Jewish population (79%), and minority Arab population (21%). The Arab minority generally live in lower socioeconomic areas, with more crowded housing and have higher rates of unemployment [8]. Healthcare is available to all through the public health system.

Israeli communities from lower socioeconomic groups and higher neighborhood density demonstrated higher COVID-19 morbidity [9]. During February–October 2020, there were 6.3 times more confirmed cases per million population in the lowest, compared with the highest SES groups; 3.8 times more hospitalized patients and 2.8 times more deaths in the lowest, compared with the highest socioeconomic category [10]. These inequalities in viral spread and disease severity raise the importance of equal distribution of the COVID-19 vaccines, both in order to reduce nationwide morbidity and to prevent widening of disparities.

This study aimed to examine COVID-19 vaccination rate by neighborhood socioeconomic status and ethnic group in Israel.

## Methods

### Data sources

Data were obtained from the Israeli Ministry of Health’s publicly available open COVID-19 database (https://data.gov.il/dataset/covid-19), which includes information on 268 medium or large (2000 inhabitants or more) urban localities and is updated daily. 8.214 million residents, comprising 88% of the total 9.291 million residents, were included in the analysis [5].

Details on the rate of vaccination (first, second and third doses) in Israel, by age group, were obtained from the MOH database website – Israel COVID-19 Data Tracker [11].

We linked to each locality in the MOH database to its socioeconomic category. SES categories are homogenous units on a scale of 1 (lowest) to 10 (highest) that are determined by the Central Bureau of Statistics (CBS) according to population demography, education, employment and standard of living [12]. For comparison between the Jewish and Arab populations, localities with homogenous (more than 90% of the population), either Jewish or Arab ethnic composition, were defined “Jewish” or “Arab” localities, respectively. Cities with at least 10% Arab residents are defined as “mixed cities”. 62.7, 15.6 and 21.6% of the total Israeli population reside in Jewish, Arab and mixed localities, respectively [13].

#### Data analysis

Our analysis included MOH data from December 20, 2020 (date of the first vaccination in Israel) up to August 31, 2021.

MOH data on vaccination were analyzed by SE categories and ethnicity.

Correlation between rate of vaccination and SES category of each locality (scale 1–10) was analyzed, using a Pearson correlation coefficient. Analysis also included vaccination rate and difference between the first and second vaccine dose, by neighborhood SES and ethnicity. Correlation was also assessed between vaccination and SES within each ethnic group separately. Vaccination uptake by age and ethnic group was also analysed.

## Results

**Fig. 1:**
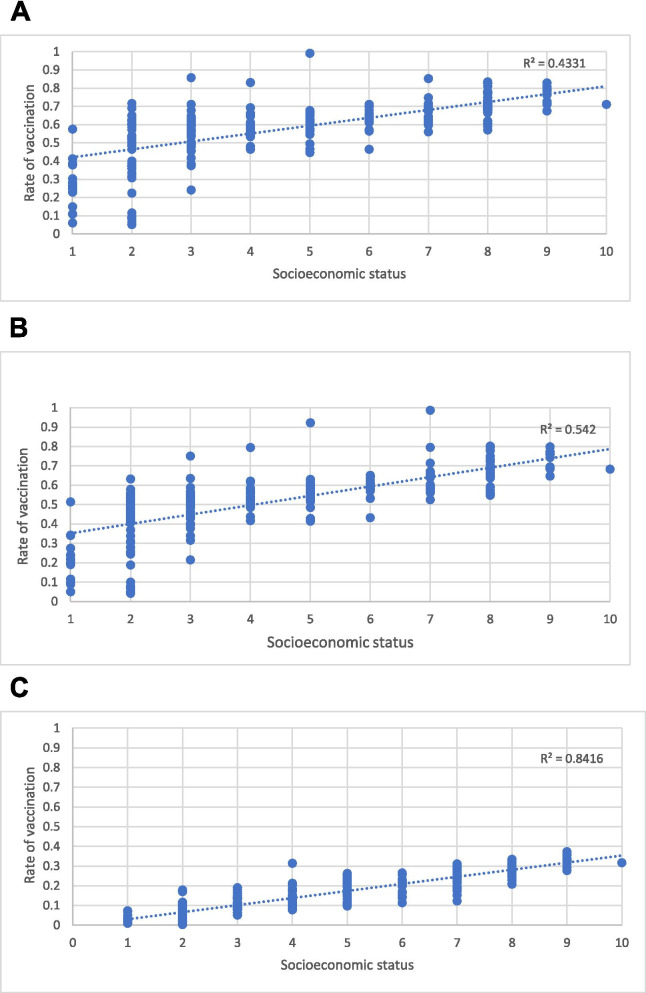
Correlation between COVID-19 vaccine uptake and SES category for each of the 268 localities,, comprising 8.214 million residents. First (**A**), second (**B**) and third (**C**) vaccine doses

A clear correlation between vaccination rate and locality’s socioeconomic category was seen, with higher vaccination rates in the higher SES categories (Fig. 1). A strong correlation (r = 0.66, *p* < .001) was demonstrated for the first dose, which was even stronger for the second (r = 0.74, p < .001) and third doses (r = 0.92, p < .001).

**Fig. 2:**
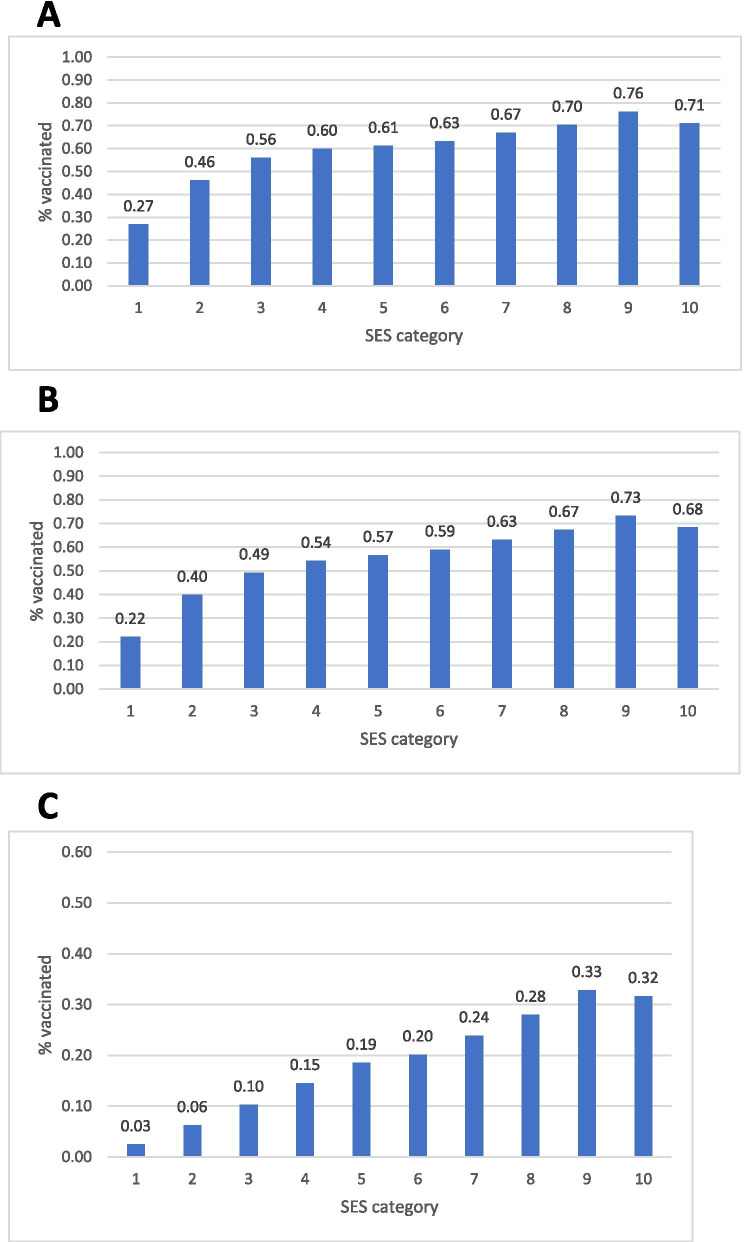
Percent of population vaccinated with the COVID-19 vaccine by SES category of residents’ locality, for the first (**A**), second (**B**) and third (**C**) vaccine doses

Vaccination coverage gradually increased with SES, with the strongest localities achieving around 70% uptake for the first and second vaccine doses, respectively, and the lowest SES communities showing the lowest rate of vaccination, with both first (27%) and second doses (22%). (Fig. 2A and B) Though vaccination with the third booster is still underway, socioeconomic differences are already clear, with over 30% vaccinated in the top two SES groups, versus 3% in the lowest SES category.

While the gradient is clear along the entire socioeconomic scale, the lowest SES localities (1 on the 1–10 scale) show a dramatic difference from the other 9 groups. The rate ratio between SES category 10 and category 1 is 2.63 and 3.09 for the first and second vaccinations, respectively (Fig. 2). More than half of individuals in SES groups 1–3 have not yet received the second vaccine dose.

**Fig. 3:**
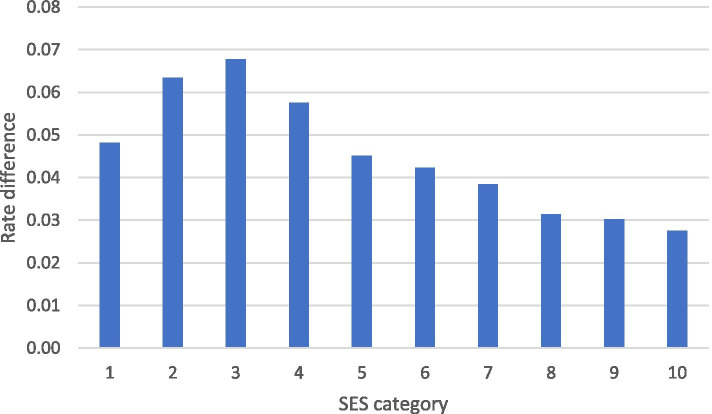
Rate difference between first and second vaccine doses, by SES category of locality

Figure 3 presents rate difference between uptake of the first and second vaccine doses, by SES category. Except for the lowest SE category, the rate difference decreases progressively as SES increases, i.e. socioeconomic disparities are more prominent in the second, compared with the first vaccine dose.

**Fig. 4:**
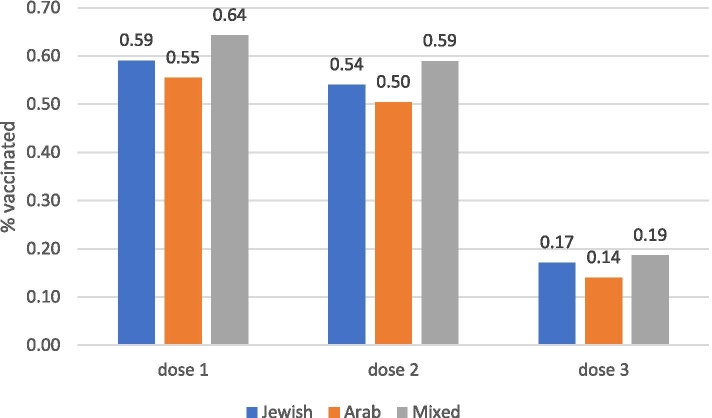
Percent of population vaccinated with first, second and third doses by ethnic group of locality (Arab, Jewish and mixed localities)

Figure 4 shows higher rates of COVID vaccination in Jewish and mixed localities, and lower vaccination in the Arab population. Rate ratio for vaccination uptake between the Jewish and Arab localities was 1.07 and 1.08 for the first and second doses, respectively.

Within each ethnic group, correlations with SES were examined (to control for the fact that Arab localities belong to lower SES categories). The socioeconomic gradient was apparent in both Jewish, Arab and mixed localities (Table 1).

**Table 1:** Correlation coefficients between vaccination and SES by ethnic group

	Jewish	Arab	Mixed
First dose	0.66	0.65	0.77
Second dose	0.74	0.72	0.89
Third dose	0.93	0.88	0.87

We further analysed vaccine uptake by age group and ethnicity, since the Arab population is generally younger, with a younger mean age than the Jewish population – uptake was higher in the Jewish population both for under and over 60s, for all doses. The disparity was greater for younger people than for older over 60s (33% difference for first dose under 60s vs 22% for over 60s; 37% vs 26% for second dose; 75% vs 49% for third dose) (not shown).

## Discussion

Despite having a public healthcare system, and making COVID-19 vaccines freely available to the whole population aged 16 and over, results demonstrate considerable socioeconomic and ethnic differences in rates of vaccination in Israel.

In line with the widely studied socioeconomic gradient in health [14], these findings demonstrate a clear gradient in COVID-19 vaccination, with significantly lower rates in the poorest sectors of society, rising to the highest rates at the highest levels of the socioeconomic ladder.

Both socioeconomic and ethnic disparities were larger for the second, compared with the first vaccine dose. The fact that people in lower SES categories were less likely to have completed their second dose by the time of analysis, could be due to going later to get their first dose, or missing the second dose.

Vaccination uptake was lower in Arab compared to Jewish areas. Socioeconomic gradient in vaccine uptake was also demonstrated within the Arab ethnic minority population. The disparity between Jewish and Arab populations in vaccination was greater for younger age groups.

### How do these findings compare with preliminary international and national reports?

A growing body of evidence has shown that lower SES and minority communities are at greater risk of severe COVID-19 morbidity and mortality, making vaccination crucial in these groups [1–3].

An Australian survey in April 2020 found that inadequate health literacy and lower education level were significantly associated with a reluctance to be vaccinated against both influenza and COVID-19 [15]. A poll conducted in the UK by the Royal Society for Public Health during December 2020 demonstrated that acceptance of the COVID vaccine was significantly lower in ethnic minorities (57% compared to 79% in white respondents) and in low SES groups (70% acceptance among lowest earners, compared to 84% among highest earners) [16].

Among 67,000 adults surveyed in the US during January 2021, both education and pre-pandemic income levels demonstrated positive dose-response with vaccine initiation (uptake of ≥1 vaccine dose). Considerable financial difficulties were linked to 44% lower odds of vaccination, and vaccine uptake was lower among Black respondents [17]. The most common reasons for vaccine hesitancy were concerns about side effects and safety [17]. Furthermore, significantly lower COVID-19 vaccination rates were demonstrated among ethnic minority healthcare workers in the UK (70.9% in white workers v 58.5% in South Asian and 36.8% in black workers) [18].

### What could explain the socioeconomic and ethnic gap in COVID vaccination?

The economic toll of the pandemic hit weaker populations harder. This might explain hesitancy in taking preventive health actions, and greater fear of side effects that might further decrease earning capabilities.

The Arab ethnic minority in Israel differs culturally and religiously from the Jewish majority, living typically in lower SES areas, more than half of them in the geographic periphery, and demonstrating poorer health outcomes compared with the general population [8]. Despite being hit harder especially in the second and third waves of the pandemic, vaccination uptake was low, which could be partly explained by a lower level of trust in governmental institutions, compared with the general population [19]. This pattern contrasts with routine childhood vaccinations in Israel, where rates are higher in the Arab compared to the Jewish population [20], but compares to research around the world where lower immunization rates were demonstrated in ethnic minorities and in low SES groups [21, 22].

Localities ranked with the lowest SES level of 1 which demonstrated significantly lower rate of vaccination, comprise harder to reach minority groups, including many Bedouin towns, and some ultra-Orthodox localities. Several reasons for non- or delayed vaccination in the lower SES groups may include religious beliefs, poorer health literacy and logistical difficulties. At the beginning of the vaccination campaign, due to complex logistics, most vaccination sites were located in larger urban cities; subsequently mobile vaccination venues were put in place, to broaden access throughout the country. This might have created a lag contributing to socioeconomic and ethnic disparities. Public health messages may not have been optimally culturally adapted, alongside less use of digital media in these communities, and less familiarity or relevance of the incentive scheme implemented by the Israeli authorities (the “green pass”) [7].

Lower health literacy among people from lower SES backgrounds [23], might explain less exposure to, or less understanding of the messages conveyed via different media platforms. Difficulties in understanding health information surrounding COVID vaccination (which is not always clearly presented in lay language) might raise fears of vaccine side effects and long term health implications, which are among the leading reasons for vaccine hesitancy.

### First dose vs second dose

Several factors may explain why lower SES groups were even less likely to complete vaccination with the second dose. Side effects, like headache, fever and flu-like symptoms, were reported more frequently after the second, compared with the first dose [24]. People with lower trust in the authorities might have dropped out of the vaccination scheme after the first dose, which offers if not optimal, at least some protection. Fear of long term effects of the second vaccine, that might worsen an already fragile financial status, may have affected the decision to comply, as well as practical issues of access, missing work etc. to attend the appointment (particularly for manual workers – 45% of men in the Arab sector) [25]. Furthermore, it should be noted that by the time of the analysis some people who delayed the first dose may not yet have completed the 3-week-interval between the two doses. Since vaccination with the third (booster) dose is still underway, it is too early to analyze whether disparities have widened compared with the previous doses.

Despite universal health coverage and substantial improvements in overall population health, Israel demonstrates considerable ethnic, socioeconomic and geographic disparities in health behaviors and prevalence of non-communicable diseases [26]. While all Israeli citizens have access to healthcare, the lowest SES groups, which include many Arab localities, have poorer infrastructure and access to facilities. Despite all outreach efforts, COVID-19 vaccination rates remain much lower than other groups in these areas.

Several strategies have been implemented in an attempt to raise vaccination rates, including outreach to communities with low rates, recruiting opinion leaders, public vaccination of senior officials acting as role models, and the use of ‘immunity passports’. Vaccine pop-up centers were established throughout the country for maximal access [27]. The “green pass” incentive scheme only came into effect 2 months into the vaccination program, but was publicly discussed from the beginning of the operation. It might have exerted a differential effect on the weakest populations, including low SES and minority groups, since the economic effects of the pandemic left many unemployed or with less stable financial status, therefore while it may help persuade more affluent communities, entrance to cultural and sports events or restaurants might not be perceived as a relevant incentive.

In parallel to the socioeconomic gradient in vaccination seen within Israel, limited access to vaccines around the world has led to vaccine inequity, with richer countries having greater access and able to inoculate their population much faster than poorer countries. It is particularly important to reach low SES groups and those at higher risk of severe COVID disease. While minority and low SES groups continue to lag behind in vaccination, they will continue to suffer disproportionately from COVID. Efforts should be made for more equitable vaccine distribution, and ways of targeting low SES groups, as well as identifying barriers to vaccination and making accurate culturally-sensitive information widely available.

### Limitations

Several limitations should be considered. Individual data was not available and locality was used as a proxy for ethnic group and SES. The two most deprived populations, the ultra-orthodox and the Arab populations, have younger mean age compared with the overall Israeli population, which might affect the interpretation of our findings. However, to date the vaccine is approved for people aged 12 and over, therefore the effect of younger populations is probably smaller.

## Conclusion

Israel has been a global leader in COVID vaccination. Analysis of national vaccination data by sociodemographic characteristics highlights considerable socioeconomic and ethnic disparities in the uptake of vaccines in a high-income country with a public health system.

With many people still undecided about whether they will take the COVID vaccine [28], it is important to identify those who are hesitant. Strategies to address the imbalance in vaccine uptake will need to identify barriers, provide targeted information, and include trust-building in disadvantaged communities, as well as providing clear and easy to understand information on side effects and on the relative risks of the vaccine and of the disease itself. Incentive strategies, if implemented, should be adapted to be relevant to all of the population.

The results of this analysis of a nation-wide vaccination operation are a call for action to address the socioeconomic gradient in COVID vaccination, and focus efforts on helping those hit hardest by the health and economic tolls of the pandemic to obtain immunization and thus improve their health horizons and promote health equity.

## Data Availability

All data available in the Ministry of health, Israel website- URL: https://govextra.gov.il/ministry-of-health/corona/corona-virus-en/

## Abbreviation

COVID-19: Corona Virus December 2019
